# Clinical Characteristics and Prognostic Factors of Testicular Sarcoma: A Population-Based Study

**DOI:** 10.3389/fonc.2021.614093

**Published:** 2021-02-25

**Authors:** Xingyuan Wang, Zeyu Chen, Shi Qiu, Dehong Cao, Kun Jin, Jin Li, Bo Chen, Haoran Lei, Yin Huang, Yige Bao, Lu Yang, Liangren Liu, Qiang Wei

**Affiliations:** ^1^ Department of Urology, Institute of Urology, West China Hospital, Sichuan University, Chengdu, China; ^2^ West China School of Medicine, Sichuan University, Chengdu, China; ^3^ Center of Biomedical Big Data, West China Hospital, Sichuan University, Chengdu, China

**Keywords:** testicular sarcoma, prognosis, cancer-specific survival, metastasis, differentiation grade

## Abstract

**Objectives:**

To study clinical characteristics and factors that may affect the prognosis of testicular sarcoma patients.

**Patients and Methods:**

In the Surveillance Epidemiology and End Results database (2006–2016), people with testicular sarcoma were enrolled in our research. Multivariable Cox proportional hazard model and Multivariable Logistic regression model were used to compare the impact of different factors on cancer-specific survival, localized metastasis, and distant metastasis.

**Results:**

This research was based on the registry information of 158 testicular sarcoma patients. All patients with a median age of 17.00 (1.00–93.00) years were pathologically diagnosed with orchiectomy or needle biopsy specimens. Patients with Grade I, II, III, and IV testicular sarcoma accounted for 34.29% (n = 24), 10.10% (n = 7), 22.86% (n = 16), and 32.86% (n = 23) of all patients, respectively. There were 42 (30.43%), 53 (38.41%), 15 (10.87%), 20 (14.49%), 5 (3.62%), 3 (2.17%) patients with Tis, T1, T2, T3, T4, and >T4 (the invasion degree exceeded the staging system of testicular cancer) disease respectively. Among all included patients, localized metastasis occurred in 31 (20.13%) patients, distant metastasis was found in 28 (18.18%) patients during observation, and 61.69% (n = 95) had no metastasis. Thirty-two (20.25%) patients died of this cancer. According to our study, patients with distant metastasis [OR = 17.86, 95% CI (4.63–68.84), p < 0.0001] and T3 disease [OR = 4.13, 95% CI (1.10–15.53), p = 0.0359] were more likely to die of this cancer. Patients with advanced T stage were more likely to occur distant metastasis, [OR = 13.91, 95% CI (1.80–107.54), p = 0.0116] for T3 and [OR = 16.36, 95% CI (1.36–196.21), p = 0.0275] for T4.

**Conclusions:**

According to our research, factors including metastasis and higher T stage were significantly related with poorer prognosis of testicular sarcoma. Higher T stage was also found to be a risk factor of distant metastasis. The recognization of these poor prognostic factors may allow physicians to make comprehensive and appropriate management decision for testicular sarcoma patients.

## Introduction

Sarcomas are a heterogeneous group of malignant tumors of mesenchymal origin (1–3). They are relatively rare, representing less than 1% of all adult malignancies and 12% of pediatric malignancies (1, 2). Sarcomas can occur at all anatomic sites in the body, but the majority are in the extremities (2). A study reviewed the anatomic distribution of soft tissue sarcomas in 4,550 adults, 46% of them occurred at thigh, buttock, and groin, 13% occurred at upper extremity, torso and retroperitoneum accounted for 18% and 13% respectively, while head and neck only constituted 9% (4). The care for patients with sarcomas is usually based on anatomic site and histology, so there is no unified treatment standard.

The majority of primary testicular tumors derive from germ cell origin. Primary testicular sarcoma (TS) is particularly rare. Since it differs from primary testicular germ cell tumors (TGCT), the risk assessment system currently used for testicular tumors shall not be applicable for TS.

As far as we know, a number of cases of TS have been reported in the previous literature (5–18), but no cohort study has been published. This study aimed to identify the clinical characteristics and factors that may affect the prognosis of TS patients. This study was based on information from the National Cancer Institute’s Surveillance Epidemiology and End Results (SEER, RRID: SCR_006902) database (2006–2016).

## Materials and Methods

### Study Population

We identified men with TS from 2006 to 2016 from cancer registries captured by the SEER Program. According to the International Classification of Disease for Oncology, Third Edition (ICD-O-3), the histology was restricted to the sarcoma including ICD-O-3 8800/3 (Sarcoma, NOS), ICD-O-3 8802/3 (Giant cell sarcoma), ICD-O-3 8811/3 (Fibromyxosarcoma), ICD-O-3 8830/3 (Malignant fibrous histiocytoma), ICD-O-3 8850/3 (Liposarcoma, NOS), ICD-O-3 8851/3 (Liposarcoma, well differentiated), ICD-O-3 8858/3 (Differentiated liposarcoma), ICD-O-3 8890/3 (Leiomyosarcoma, NOS), ICD-O-3 8895/3 (Myosarcoma), ICD-O-3 8900/3 (Rhabdomyosarcoma, NOS), ICD-O-3 8901/3 (Pleomorphic rhabdomyosarcoma, adult type), ICD-O-3 8902/3 (Mixed type rhabdomyosarcoma), ICD-O-3 8910/3 (embryonal rhabdomyosarcoma, NOS), ICD-O-3 8912/3 (Spindle cell rhabdomyosarcoma), ICD-O-3 8920/3 (Alveolar rhabdomyosarcoma), and ICD-O-3 8921/3 (Rhabdomyosarcoma with ganglionic differentiation). Tumors containing elements other than sarcoma and patients with neoplasms in other sites were excluded. The primary cancer site was restricted to the testis (C62). Sarcomas from other sites that metastasized to the testicle were also excluded. Finally 158 patients were enrolled in our study. Data we obtained from the database contained age, survival time, region, race, marital status, year of diagnosis, differentiation grade, laterality, tumor size, surgery, serum tumor markers [Alpha Fetoprotein (AFP), human Chorionic Gonadotropin (hCG), Lactate Dehydrogenase (LDH)], stage, and cancer-specific mortality (CSM).

### Independent Variable and Endpoint

The endpoints of this study included cancer-specific survival (CSS), localized metastasis (LM), and distant metastasis (DM). LM referred to regional lymph nodes metastasis. We compared the impact of different independent factors on them, including age at diagnosis, differentiation grade, laterality, tumor size, surgery, serum tumor markers, metastasis, and T stage.

### Statistical Analysis

Firstly, we assessed the distribution of baseline characteristics with the use of a two‐sample t test and a chi-square test to compare continuous and categorical variables, respectively. Data were presented as median (interquartile range or minimum value–maximum value) for continuous variables and as frequency (%) for categorical variables.

Secondly, Kaplan-Meier survival estimate was used to compare survival of patients of different differentiation grades.

Thirdly, multivariable cox proportional hazard model and multivariable logistic regression model were used to compare the impact of different factors on CSS, LM, and DM, with adjustment for region, race, marital status, and year of diagnosis.

All statistical analyses were performed using Empower Stats 2.0. A *P* value <0.05 was considered statistically significant.

## Results

### Baseline Characteristics

A total of 158 TS patients were included in our study. All patients were diagnosed pathologically through surgery or needle biopsy. The patients were of a median age of 17 (interquartile range, 7.25–61.50) years. Patients with Grade I, II, III, and IV testicular sarcoma accounted for 34.29% (n = 24), 10.10% (n = 7), 22.86% (n = 16), and 32.86% (n = 23) of all patients, respectively. There were 42 (30.43%), 53 (38.41%), 15 (10.87%), 20 (14.49%), 5 (3.62%), 3 (2.17%) patients with Tis, T1, T2, T3, T4, and >T4 (the invasion degree exceeded the staging system of testicular cancer) disease respectively. Among all included patients, localized metastasis occurred in 31 (20.13%) patients, distant metastasis was found in 28 (18.18%) patients during observation. Details of the baseline information was tabulated in **Table 1**.

**Table 1:** Baseline characteristics of all TS patients.

**Age at diagnosis, Median (Q1-Q3)**	17.00 (7.25–61.50)
**Region, N (%)**	
Pacific Coast	76 (48.10%)
East	57 (36.08%)
Northern Plains	14 (8.86%)
Southwest	11 (6.96%)
**Race, N (%)**	
White	117 (76.47%)
Black	28 (18.30%)
Asian or Pacific Islander	7 (4.58%)
American Indian/Alaska Native	1 (0.65%)
**Marital status, N (%)**	
Single (never married)/Unmarried or Domestic Partner	104 (69.80%)
Married (including common law)	38 (25.50%)
Divorced/separated/widowed	7 (4.70%)
**Year of diagnosis, N (%)**	
2006	12 (7.59%)
2007	12 (7.59%)
2008	22 (13.92%)
2009	9 (5.70%)
2010	10 (6.33%)
2011	20 (12.66%)
2012	15 (9.49%)
2013	13 (8.23%)
2014	16 (10.13%)
2015	13 (8.23%)
2016	16 (10.13%)
**Differentiation grade, N (%)**	
Well differentiated; Grade I (G1)	24 (34.29%)
Moderately differentiated; Grade II (G2)	7 (10.00%)
Poorly differentiated; Grade III (G3)	16 (22.86%)
Undifferentiated; anaplastic; Grade IV (G4)	23 (32.86%)
**Laterality, N (%)**	
Unilateral	75 (47.47%)
Bilateral	83 (52.53%)
**Tumor size, N (%)**	
<2.0	4 (3.25%)
2.0–4.0	20 (16.26%)
≥4.0	99 (80.49%)
**Surgery, N (%)**	
None	2 (1.27%)
Partial dissection	1 (0.63%)
Orchiectomy	153 (96.84%)
Method unknown	2 (1.27%)
**RPLND, N (%)**	
Yes	36 (23.08%)
No	120 (76.92%)
**Radiotherapy, N (%)**	
None	117 (74.05%)
Adjuvant	39 (24.68%)
Neoadjuvant	2 (1.27%)
**AFP, N (%)**	
Normal	20 (83.33%)
Normal–1,000	2 (8.33%)
1,000–10,000	1 (4.17%)
>10,000	1 (4.17%)
**hCG, N (%)**	
Normal	18 (78.26%)
Normal–5,000	5 (21.74%)
**LDH, N (%)**	
Normal	11 (57.89%)
Normal–1.5N^a^	5 (26.32%)
1.5N - 10N	3 (15.79%)
**Metastasis, N (%)**	
No	95 (61.69%)
Localized	31 (20.13%)
Distant	28 (18.18%)
**Invasion, N (%)**	
Tis	42 (30.43%)
T1	53 (38.41%)
T2	15 (10.87%)
T3	20 (14.49%)
T4	5 (3.62%)
>T4	3 (2.17%)

Q, quartile; RPLND, retroperitoneal lymph node dissection; AFP, Alpha Fetoprotein; hCG, human Chorionic Gonadotropin; LDH, Lactate Dehydrogenase.

a N, normal.

### Survival Data

**Figure 1:**
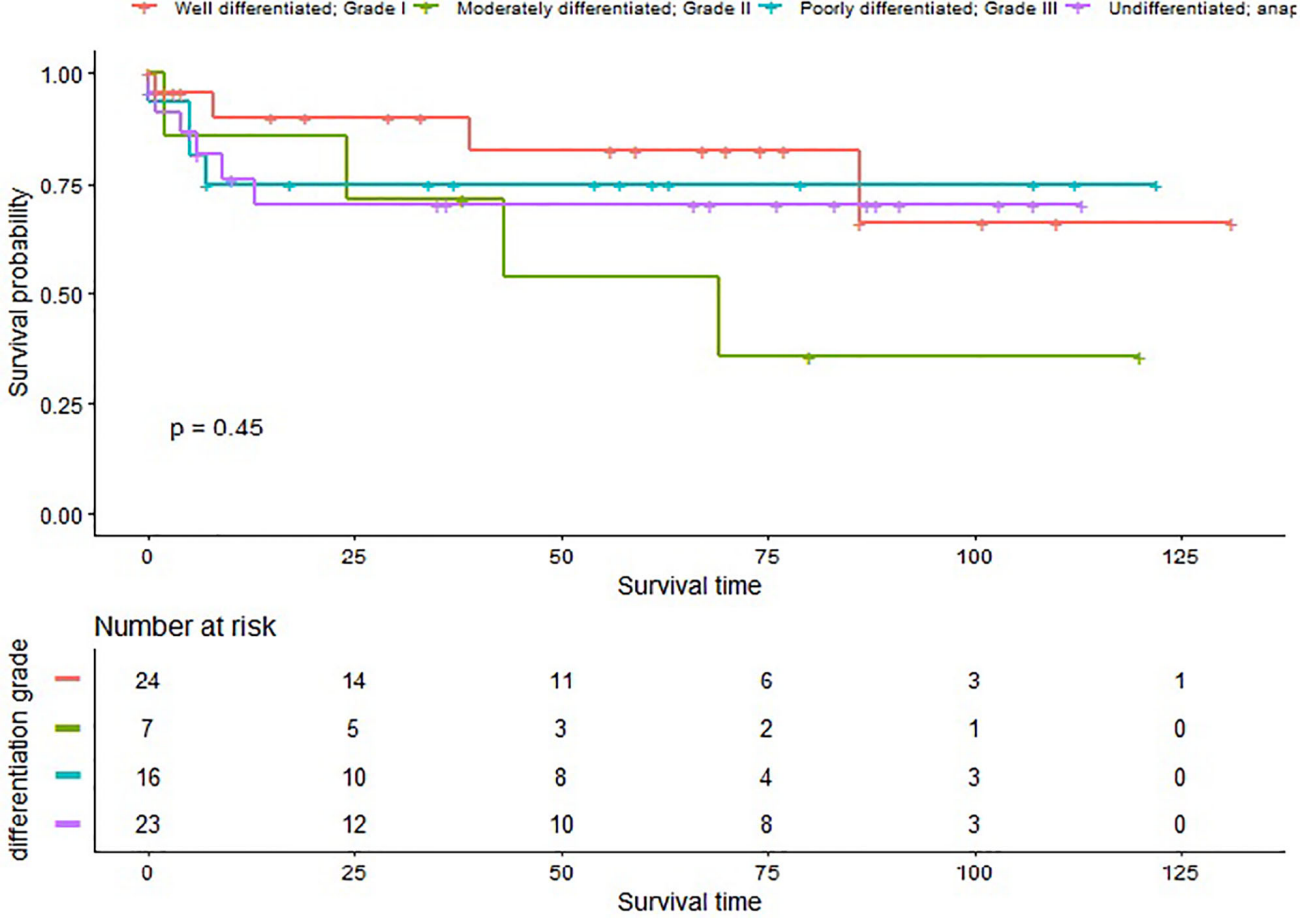
Cancer specific survival in patients with different differentiation grades.

In our study, 32 (20.25%) patients died of this cancer, with a median survival time of 43.00 (0.00–131.00) months. The 1- and 3-year CSS rate of G1, G2, G3, and G4 patients was 90.03% (95% CI 77.69–100.00%), 85.71% (63.34–100.00%), 75.00% (56.52–99.52%), 76.02% (59.60–96.98%), and 82.52% (65.87–100.00%), 71.43% (44.71–100.00%), 75% (56.52–99.52%), 70.17% (52.53–93.75%), respectively (**Figure 1**).

### Risk Factors

Cox proportional hazard model was used to evaluate the impact of several factors on CSM. According to our study, patients with distant metastasis [OR = 17.86, 95% CI (4.63–68.84), p < 0.0001] and T3 disease [OR = 4.13, 95% CI (1.10–15.53), p = 0.0359] were more likely to die of this cancer. However, we did not find statistical difference in the risk of CSM between different differentiation grade, laterality, tumor size, surgery, AFP, hCG, LDH (p > 0.05) (**Table 2**).

**Table 2:** CSM risk factors in all patients.

Exposure	Univariable model HR (95% CI) p-value	Multivariable model HR (95% CI) p-value
**Age at diagnosis**	1.02 (1.01, 1.03) 0.0003^*^	1.06 (1.03, 1.09) 0.0002^*^
**Differentiation grade**		
G1	1.0	1.0
G2	2.97 (0.74, 11.90) 0.1239	inf. (0.00, Inf) 0.9980
G3	1.42 (0.36, 5.70) 0.6169	0.28 (0.02, 4.09) 0.3525
G4	1.56 (0.44, 5.54) 0.4909	1.91 (0.34, 10.78) 0.4658
**Laterality**		
Unilateral	1.0	1.0
Bilateral	0.81 (0.40, 1.61) 0.5409	0.92 (0.38, 2.21) 0.8465
**Tumor size**		
<4 cm	1.0	1.0
≥4 cm	2.05 (0.61, 6.83) 0.2429	2.13 (0.52, 8.70) 0.2902
**Surgery**		
None	1.0	1.0
Partial dissection	1.00 (0.00, Inf) 1.0000	0.27 (0.00, Inf) 1.0000
Orchiectomy	inf. (0.00, Inf) 0.9982	inf. (0.00, Inf) 0.9993
Method unknown	1.00 (0.00, Inf) 1.0000	1.00 (0.00, Inf) 1.0000
**Radiotherapy**		
None	1.0	1.0
Adjuvant	1.52 (0.73, 3.15) 0.2606	3.91 (1.42, 10.76) 0.0081^*^
Neoadjuvant	0.00 (0.00, Inf) 0.9968	0.00 (0.00, Inf) 0.9989
**AFP**		
Normal	1.0	1.0
Normal–1,000	0.00 (0.00, Inf) 0.9993	0.00 (0.00, Inf) 0.9999
1,000–10,000	0.00 (0.00, Inf) 0.9995	0.02 (0.00, Inf) 1.0000
**hCG**		
Normal	1.0	1.0
Normal - 5000	3.28 (0.54, 19.79) 0.1948	6.81 (0.36, 129.27) 0.2013
**LDH**		
Normal	1.0	1.0
Normal–1.5N^a^	7.45 (0.77, 72.22) 0.0831	1.0
1.5N–10N	0.00 (0.00, Inf) 0.9991	1.0
**Metastasis**		
No	1.0	1.0
Localized	1.28 (0.45, 3.64) 0.6410	1.61 (0.37, 7.05) 0.5267
Distant	4.97 (2.32, 10.64) <0.0001^*^	17.86 (4.63, 68.84) <0.0001^*^
**Invasion**		
Tis	1.0	1.0
T1	0.60 (0.23, 1.55) 0.2866	0.42 (0.14, 1.23) 0.1130
T2	1.36 (0.42, 4.42) 0.6085	2.27 (0.59, 8.73) 0.2336
T3	1.89 (0.70, 5.08) 0.2088	4.13 (1.10, 15.53) 0.0359^*^
T4	0.97 (0.12, 7.63) 0.9739	2.46 (0.22, 27.48) 0.4655
>T4	1.41 (0.18, 11.15) 0.7473	0.00 (0.00, Inf) 0.9989

AFP, Alpha Fetoprotein; hCG, human Chorionic Gonadotropin; LDH, Lactate Dehydrogenase.

a N, normal.

*Statistically significant.

Radiotherapy was found to be related with higher risk of CSM after adjusting for marital status, race, region, and year of diagnosis. Hence, we compared baseline characteristics of patients received adjuvant radiotherapy with those not. Significant difference was found in differentiation grade, status of metastasis, and T stage (**Supplementary Table 1**). After adjusting for marital status, race, region, year of diagnosis, differentiation grade, status of metastasis, and T stage, that association was not statistically significant [34407.88 (0.00, Inf) 0.9960].

We also used multivariable logistic regression analysis to evaluate the impact of several factors on LM. Patients with Grade III disease had a higher risk of localized metastasis [OR = 14.29, 95% CI (1.41–144.36), p = 0.0242] before adjustment. However, the difference became not statistically significant after adjusting for other variates [OR = 83.01, 95% CI (0.61, 11366.93), P = 0.0783]. There were either no statistical significance in the impact of laterality, tumor size, T stage on LM (p > 0.05) (**Table 3**).

**Table 3:** LM risk factors in all patients.

Exposure	Univariable model HR (95% CI) p-value	Multivariable model HR (95% CI) p-value
**Age at diagnosis**	0.99 (0.97, 1.00) 0.1458	1.01 (0.96, 1.05) 0.7963
**Differentiation grade**		
G1	1.0	1.0
G2	3.33 (0.18, 61.68) 0.4187	0.00 (0.00, Inf) 0.9980
G3	14.29 (1.41, 144.36) 0.0242^*^	83.01 (0.61, 11366.93) 0.0783
G4	2.86 (0.24, 34.66) 0.4097	8.39 (0.13, 538.41) 0.3165
**Laterality**		
Unilateral	1.0	1.0
Bilateral	1.69 (0.74, 3.86) 0.2155	1.43 (0.51, 3.97) 0.4939
**Invasion**		
Tis	1.0	1.0
T1	10.50 (1.30, 84.88) 0.0274^*^	inf. (0.00, Inf) 0.9959
T2	8.75 (0.70, 108.79) 0.0916	inf. (0.00, Inf) 0.9959
T3	inf. (0.00, Inf) 0.9910	inf. (0.00, Inf) 0.9948
T4	inf. (0.00, Inf) 0.9962	inf. (0.00, Inf) 0.9974
>T4	1.0	1.0
**Tumor size**		
<4 cm	1.0	1.0
≥4 cm	1.40 (0.46, 4.26) 0.5530	1.94 (0.46, 8.12) 0.3658

*Statistically significant.

Multivariable logistic regression model was used to evaluate the impact of several factors on DM. Patients with Grade IV disease had a higher risk of distant metastasis [OR = 9.19, 95% CI (1.02–82.42), p = 0.0476] before adjustment. Nonetheless, the difference became not statistically significant after adjusting for other variates [OR = 7.38, 95% CI (0.53, 102.36), P = 0.1362]. Patients with advanced T stage were of higher risk of distant metastasis [OR = 13.91, 95% CI (1.80–107.54), p = 0.0116] for T3 and [OR = 16.36, 95% CI (1.36–196.21), p = 0.0275] for T4 (**Table 4**).

**Table 4:** DM risk factors in all patients.

Exposure	Univariable model OR (95% CI) p-value	Multivariable model OR (95% CI) p-value
**Age at diagnosis**	0.99 (0.98, 1.01) 0.3828	1.07 (1.02, 1.12) 0.0028^*^
**Differentiation grade**		
G1	1.0	1.0
G2	0.00 (0.00, Inf) 0.9950	16.56 (0.00, Inf) 0.9998
G3	7.00 (0.70, 70.05) 0.0977	5.90 (0.23, 153.22) 0.2855
G4	9.19 (1.02, 82.42) 0.0476^*^	7.38 (0.53, 102.36) 0.1362
**Laterality**		
Unilateral	1.0	1.0
Bilateral	1.08 (0.48, 2.46) 0.8493	1.17 (0.43, 3.20) 0.7558
**AFP**		
Normal	1.0	1.0
Normal–1,000	3.00 (0.16, 57.37) 0.4656	inf. (0.00, Inf) 0.9994
1,000–10,000	0.00 (0.00, Inf) 0.9967	0.00 (0.00, Inf) 0.9995
>10,000	inf. (0.00, Inf) 0.9962	inf. (0.00, Inf) 0.9994
**hCG**		
Normal	1.0	1.0
Normal - 5000	3.90 (0.49, 30.76) 0.1965	inf. (0.00, Inf) 0.9983
**LDH**		
Normal	1.0	
Normal–1.5N^a^	15.00 (0.98, 228.91) 0.0515	
1.5N–10N	inf. (0.00, Inf) 0.9956	
**Invasion**		
Tis	1.0	1.0
T1	0.12 (0.01, 1.00) 0.0500	0.08 (0.01, 0.93) 0.0432
T2	3.00 (0.76, 11.90) 0.1182	3.74 (0.55, 25.40) 0.1766
T3	4.91 (1.43, 16.86) 0.0115^*^	13.91 (1.80, 107.54) 0.0116^*^
T4	9.00 (1.23, 65.64) 0.0302^*^	16.36 (1.36, 196.21) 0.0275^*^
>T4	inf. (0.00, Inf) 0.9894	inf. (0.00, Inf) 0.9978
**Tumor size**		
<4 cm	1.0	1.0
≥4 cm	7.36 (0.94, 57.39) 0.0568	8.87 (0.92, 85.27) 0.0587

AFP, Alpha Fetoprotein; hCG, human Chorionic Gonadotropin; LDH, Lactate Dehydrogenase.

a N, normal.

*Statistically significant.

## Discussion

Sarcomas are complicated malignancies encompassing a broad histopathologic spectrum. Approximately 80 percent of new cases of sarcomas originate from soft tissue, and the rest originate from bone (1, 2). As classified by the fourth edition of the World Health Organization (WHO), there are more than 100 different histologic subtypes of soft-tissue neoplasms (3, 19). Histologic grade is currently recognized the most important prognostic factor for sarcomas and is predictive of distant metastasis and cancer-specific survival (2, 3). There are several grading systems. The AJCC Staging System uses a four-grade scheme ranging from G1 (well-differentiated) to G4 (undifferentiated) (20). The French Federation of Cancer Centers Sarcoma Group (FNCLCC) grading schema, basing on three parameters: differentiation, mitotic rate, and necrosis, is widely recommended (2, 20, 21). In addition to histologic grade, tumor size and pathologic stage at the time of diagnosis are counted as another two most important prognostic factors (2).

Due to the histological difference between TS and TGCT, the clinical and pathological characteristic of TS may differ from TGCT. The management decision of TS should not be made on the basis of current guidelines for testicular tumors (which was mainly on TGCTs). Since TS is particularly rare, studies about this cancer are limited, and most of them are case reports or case reviews (5–18). To our knowledge, this is the first cohort study based on a relatively large amount of TS patients, reporting clinical characteristics and factors that may affect the prognosis of TS patients.

As reported in our study, patients with distant metastasis had a 17.86 fold increased risk to die of TS itself [OR = 17.86, 95% CI (4.63–68.84), p < 0.0001], and patients with T3 disease had a 4.13 fold increased risk [OR = 4.13, 95% CI (1.10–15.53), p = 0.0359]. The risk of DM in patients with advanced T stage was higher than those with lower T stage, 13.91 fold (95% CI, 1.80 to 107.54 fold) for T3 and 16.36 fold (95% CI, 1.36 to 196.21 fold) for T4. We can conclude that necessary imaging techniques should be used to evaluate the extent of a primary tumor and to establish the presence or absence of metastatic disease. For those patients with metastasis or advanced T stage disease, further treatment and comprehensive management should be formulated. At present there are no relevant guidelines regarding retroperitoneal lymph node dissection (RPLND) in TS patients. For retroperitoneal sarcomas, the cornerstone of treatment is surgical resection (3). So we consider RPLND necessary when imaging shows enlarged retroperitoneal lymph nodes.

Patients with Grade II disease had a 14.29 fold increased risk of LM [OR = 14.29, 95% CI (1.41–144.36), p = 0.0242], and patients with Grade IV disease had a 9.19 fold increased risk of DM [OR = 9.19, 95% CI (1.02–82.42), p = 0.0476] before adjustment. However the difference became not statistically significant after adjustment. In addition, we did not find there were statistical difference when evaluating the impact of differentiation grade and surgery on CSM, the impact of tumor size on CSM, LM, and DM. These findings may be due to the small sample size and the variability in the number of patients of different T stages and differentiation grades. We can still speculate from the Kaplan-Meier curve that the survival rate of TS patients lowered with worse differentiation, though this difference was not statistically significant (p > 0.05).

What’s more, most patients enrolled in our study had normal AFP, hCG, and LDH. This is consistent with previously reported cases, including but not limited to a 27-year-old male with high-grade primary testicular leimyosarcoma reported by Fouzia Siraj et al. (5) and a 51-year-old patient with primary testicular sarcoma reported by Allaway M. et al. (6). We did not find that these serum tumor markers had a statistically significant impact on CSM and DM in TS patients. Unlike in germ cell tumors, where elevations of tumor markers (AFP, hCG, and LDH) allow for staging and determination of treatment regimen, no known marker exists in TS. Future work may elucidate a prognostic biomarker for TS.

Although this is the first cohort study in TS, there are limitations of our study. Due to its retrospective nature, not all clinical data were collected in SEER database, which is a common defect in any observational study. In addition, because of the limited size of the study cohort, some outcomes were not adequately powered to detect a statistical significance. As there are no guidelines for the diagnosis and management of patients with TS, there was likely high variability in the staging and receipt of imaging as well as treatments received by the patients in this cohort.

## Conclusion

According to our study, patients with advanced T stage were more likely to have distant metastasis. Both distant metastasis and advanced T stage were associated with worse CSM. The recognization of these poor prognostic factors may allow physicians to make comprehensive and appropriate management decision for TS patients. In order to better understand this disease, larger powered studies are needed.

## Data Availability Statement

The original contributions presented in the study are included in the article/**Supplementary Material**. Further inquiries can be directed to the corresponding author.

## Ethics Statement

Ethical review and approval was not required for the study on human participants in accordance with the local legislation and institutional requirements. Written informed consent from the participants’ legal guardian/next of kin was not required to participate in this study in accordance with the national legislation and the institutional requirements.

## Author Contributions

(I) Conception and design: XW, ZC, and SQ. (II) Administrative support: YB, LY, LL, and QW. (III) Provision of study materials or patients: XW, ZC, SQ, and KJ. (IV) Collection and assembly of data: XW, ZC, JL, BC, HL, and YH. (V) Data analysis and interpretation: XW, ZC, SQ, KJ, and YB. (VI) Manuscript writing: XW, ZC, SQ, DC, KJ, JL, BC, HL, YH, YB, LY, LL, and QW. All authors contributed to the article and approved the submitted version.

## Conflict of Interest

The authors declare that the research was conducted in the absence of any commercial or financial relationships that could be construed as a potential conflict of interest.

## Supplementary Material

Supplementary Table 1 Baseline characteristics of patients received radiotherapy after surgery or not.
