# Supplementary material for: Clinical Characteristics and Prognostic Factors of Testicular Sarcoma: A Population-Based Study
Source: Front Oncol. 2021 Feb 25;11:614093. doi: 10.3389/fonc.2021.614093 (PMC7959772; doi:10.3389/fonc.2021.614093)
Supplement: Supplementary Table 1 — Baseline characteristics of patients received radiotherapy after surgery or not. [file Table_1.docx]

**Supplementary Table 1 Baseline characteristics of patients received radiotherapy after surgery or not.**

|  | **No radiotherapy** | **Adjuvant radiotherapy** | **P-value** |
| --- | --- | --- | --- |
| **N** | **117** | **39** |  |
| **Age at diagnosis, Median (Min-Max)** | 23.00 (1.00-93.00) | 16.00 (2.00-77.00) | 0.071 |
| **Survival time ^b^, Median (Min-Max)** | 43.00 (0.00-131.00) | 61.00 (0.00-123.00) | 0.693 |
| **Region, N (%)** |  |  | 0.978 |
| Pacific Coast | 56 (47.86%) | 18 (46.15%) |  |
| East | 42 (35.90%) | 15 (38.46%) |  |
| Northern Plains | 11 (9.40%) | 3 (7.69%) |  |
| Southwest | 8 (6.84%) | 3 (7.69%) |  |
| **Race, N (%)** |  |  | 0.326 |
| White | 89 (79.46%) | 26 (66.67%) |  |
| Black | 18 (16.07%) | 10 (25.64%) |  |
| Asian or Pacific Islander | 4 (3.57%) | 3 (7.69%) |  |
| American Indian/Alaska Native | 1 (0.89%) | 0 (0.00%) |  |
| **Marital status, N (%)** |  |  | 0.048* |
| Single (never married)/Unmarried or Domestic Partner | 70 (64.22%) | 32 (84.21%) |  |
| Married (including common law) | 32 (29.36%) | 6 (15.79%) |  |
| Divorced/separated/widowed | 7 (6.42%) | 0 (0.00%) |  |
| **Year of diagnosis, N (%)** |  |  | 0.229 |
| 2006 | 9 (7.69%) | 3 (7.69%) |  |
| 2007 | 8 (6.84%) | 4 (10.26%) |  |
| 2008 | 12 (10.26%) | 8 (20.51%) |  |
| 2009 | 5 (4.27%) | 4 (10.26%) |  |
| 2010 | 9 (7.69%) | 1 (2.56%) |  |
| 2011 | 12 (10.26%) | 8 (20.51%) |  |
| 2012 | 14 (11.97%) | 1 (2.56%) |  |
| 2013 | 11 (9.40%) | 2 (5.13%) |  |
| 2014 | 13 (11.11%) | 3 (7.69%) |  |
| 2015 | 11 (9.40%) | 2 (5.13%) |  |
| 2016 | 13 (11.11%) | 3 (7.69%) |  |
| **Differentiation grade, N (%)** |  |  | 0.012* |
| G1 | 22 (41.51%) | 2 (11.76%) |  |
| G2 | 6 (11.32%) | 1 (5.88%) |  |
| G3 | 13 (24.53%) | 3 (17.65%) |  |
| G4 | 12 (22.64%) | 11 (64.71%) |  |
| **Laterality, N (%)** |  |  | 0.853 |
| Unilateral | 55 (47.01%) | 19 (48.72%) |  |
| Bilateral | 62 (52.99%) | 20 (51.28%) |  |
| **Tumor size, N (%)** |  |  | 0.185 |
| <2.0 | 3 (3.37%) | 1 (3.12%) |  |
| 2.0-4.0 | 18 (20.22%) | 2 (6.25%) |  |
| ≥4.0 | 68 (76.40%) | 29 (90.62%) |  |
| **Surgery, N (%)** |  |  | 0.632 |
| None | 2 (1.71%) | 0 (0.00%) |  |
| Partial dissection | 1 (0.85%) | 0 (0.00%) |  |
| Orchiectomy | 112 (95.73%) | 39 (100.00%) |  |
| Method unknown | 2 (1.71%) | 0 (0.00%) |  |
| **AFP, N (%)** |  |  | 0.408 |
| Normal | 12 (85.71%) | 7 (87.50%) |  |
| Normal - 1000 | 1 (7.14%) | 0 (0.00%) |  |
| 1000 - 10000 | 0 (0.00%) | 1 (12.50%) |  |
| >10000 | 1 (7.14%) | 0 (0.00%) |  |
| **hCG, N (%)** |  |  | 0.586 |
| Normal | 11 (84.62%) | 6 (75.00%) |  |
| Normal - 5000 | 2 (15.38%) | 2 (25.00%) |  |
| **LDH, N (%)** |  |  | 0.155 |
| Normal | 7 (77.78%) | 3 (33.33%) |  |
| Normal - 1.5N ^a^ | 1 (11.11%) | 4 (44.44%) |  |
| 1.5N - 10N | 1 (11.11%) | 2 (22.22%) |  |
| **Metastasis, N (%)** |  |  | <0.001* |
| No | 85 (75.22%) | 9 (23.08%) |  |
| Localized | 17 (15.04%) | 14 (35.90%) |  |
| Distant | 11 (9.73%) | 16 (41.03%) |  |
| **T stage, N (%)** |  |  | <0.001* |
| Tis | 36 (36.00%) | 4 (11.11%) |  |
| T1 | 43 (43.00%) | 10 (27.78%) |  |
| T2 | 10 (10.00%) | 5 (13.89%) |  |
| T3 | 9 (9.00%) | 11 (30.56%) |  |
| T4 | 1 (1.00%) | 4 (11.11%) |  |
| >T4 | 1 (1.00%) | 2 (5.56%) |  |
| AFP = Alpha Fetoprotein.  hCG = human Chorionic Gonadotropin.  LDH = Lactate Dehydrogenase.  ^a^ N = normal.  ^b^ The survival time is indicated in months.  *statistically significant. | | | |
